# S235. MAINTENANCE TREATMENT WITH ANTIPSYCHOTIC DRUGS IN SCHIZOPHRENIA – SYSTEMATIC REVIEW AND NETWORK META-ANALYSIS

**DOI:** 10.1093/schbul/sby018.1022

**Published:** 2018-04-01

**Authors:** Johannes Schneider-Thoma, Stefan Leucht

**Affiliations:** Klinikum rechts der Isar, Technical University of Munich

## Abstract

**Background:**

Antipsychotic drugs are the mainstay of the maintenance treatment of schizophrenia and they are known to prevent psychotic relapse as compared to placebo. Nevertheless, insufficient efficacy and reoccurrence of psychotic symptoms (relapse) are frequent phenomena. Furthermore, side effects of antipsychotic drugs can be very unpleasant and even dangerous, particularly, when drugs are applied over a long time. Nowadays, many different antipsychotic substances are available and they can be used in oral or long-acting-injectable applications. However, the comparative efficacy of the different drugs in preventing relapse as well as the efficacy in specific domains of the disease (such as positive and negative symptoms) over the long term is only know in parts. Also concerning side effects it is not clear for a lot of drugs which substance should be preferred over another. The novel method of network-meta-analysis provides the possibility to use indirect evidence to compare drugs for which no direct comparison is available. Moreover, hierarchies of drugs can be created with this method, that show which drug is the best, second best, and so on, for individual outcomes.

**Methods:**

We are conducting a network-meta-analysis of randomized controlled trials (RCT) in patients with schizophrenia or schizoaffective disorder. To identify eligible studies, we searched the register of the Cochrane Schizophrenia group, the most comprehensive database of clinical trials in schizophrenia. RCTs comparing antipsychotic drugs with each other or placebo are included. We focus hereby on the clinically most important newer and older antipsychotic drugs – as identified by a survey of international schizophrenia experts. The primary outcome is patients with a psychotic relapse at any time. Relapse at specific time-points as well as several other efficacy and tolerability parameters will be evaluated as additional outcomes. We include only trials of at least 3 months in duration, conducted with patients in a stable state of the disorder. Special attention is paid to the question if populations of different studies are comparable. This is of particular importance for network-meta-analysis because this method is based on the assumption of transitivity, i.e. that each patient in the analysis would have been in principle eligible for each study in the network.

**Results:**

All so called second-generation antipsychotic drugs as well as several first-generation antipsychotics (list provided on the poster) were identified as clinically important drugs. A systematic literature search including the generic names of these drugs as well as search terms for maintenance treatment and stable condition found 3562 references. Screening of title and abstracts resulted in 1188 references referring to potentially eligible studies. In the ongoing full-text-screening and cross-referencing process 136 included studies are identified so far (complete search results presented on the poster). More detailed assessments of study characteristics are warranted to decide which studies are eligible to be included in the network-meta-analysis, i.e. to fulfill the transitivity criteria from a clinical point of view.

**Discussion:**

Studies examining antipsychotic drugs for maintenance treatment differ in inclusion criteria for participants, in criteria for stable condition and also in criteria for psychotic relapse. Therefore, some official maintenance studies may not be eligible for network-meta-analysis. However, other studies, focusing not on maintenance but on other outcomes, may fulfill the transitivity criteria. The resulting problems for conducting network-meta-analysis as well as the reasoning for inclusion and exclusion of studies will be discussed.

